# Production of alkaline pectinase: a case study investigating the use of tobacco stalk with the newly isolated strain *Bacillus tequilensis* CAS-MEI-2-33

**DOI:** 10.1186/s12896-019-0526-6

**Published:** 2019-07-12

**Authors:** Ge Zhang, Shugui Li, Yingbo Xu, Juan Wang, Fan Wang, Yuhua Xin, Zhong Shen, Haibo Zhang, Ming Ma, Haobao Liu

**Affiliations:** 1 Key Laboratory of Tobacco Biology and Processing, Tobacco Research Institute of Chinese Academy of Agricultural Sciences, Qingdao, 266101 People’s Republic of China; 2 CAS Key Laboratory of Biobased Materials, Qingdao Institute of Bioenergy and Bioprocess Technology, Chinese Academy of Sciences, Qingdao, 266101 People’s Republic of China; 3 Haikou Cigar Research Institute, Hainan Provincial Branch of CNTC, Haikou, 571100 People’s Republic of China; 4 China Tobacco Standardization Research Center, Zhengzhou Tobacco Research Institute of CNTC, Zhengzhou, 450001 People’s Republic of China; 5 Research and Development Center, China Tobacco Anhui Industrial Co., Ltd., Hefei, 230088 People’s Republic of China

**Keywords:** Alkaline pectinase, Tobacco stalk, Screening *Bacillus tequilensis*, Fermentation, Enzymatic properties, Purification

## Abstract

**Background:**

Tobacco stalk (TS), a major agricultural waste abundant in pectin, has resulted in concerns about the need for its reuse. The nicotine in TS is considered a chemical that is to\xic and hazardous to the environment.

**Results:**

In this study, *Bacillus tequilensis* CAS-MEI-2-33 was isolated from cigar wrappers to produce alkaline pectinase using TS. Subsequently, the medium and fermentation conditions for the production of pectinase by *B*. *tequilensis* CAS-MEI-2-33 were optimized. The optimal fermentation period, pH of the initial fermentation medium, concentration of TS, and inoculum amount for *B*. *tequilensis* CAS-MEI-2-33 were 40 h, 40 g/L, 7.0, and 3%, respectively. Under optimal conditions, the pectinase activity was 1370 U/mL. Then, the enzymatic properties, such as the optimum pH, reaction temperature, temperature stability, and effects of metal ions, were studied. The optimal pH was determined to be 10.0, indicating that the enzyme was an alkaline pectinase. The optimal temperature was 40 °C, and pectinase activity was stable at 40 °C. The Ag^+^ metal ions were shown to remarkably promote enzyme activity. The pectinase was partly purified by ammonium sulfate precipitation, ion exchange chromatography, and Sephacryl S-100 chromatography. Sodium dodecyl sulfate-polyacrylamide gel electrophoresis (SDS-PAGE) and LC-MS/MS analyses were utilized to analyze the pectinase.

**Conclusions:**

This study provided a new alkaline pectinase candidate and a new strategy for the use of TS.

**Electronic supplementary material:**

The online version of this article (10.1186/s12896-019-0526-6) contains supplementary material, which is available to authorized users.

## Background

Extremely large quantities of agro-industrial waste residues are generated from the processing of raw materials, such as tobacco stalk (TS), which is an abundant crop residue in China. It has been estimated that more than one hundred thousand metric tons of TS are discarded annually in China ([1] #1136). Stricter environmental legislation for tobacco industries in China has forced these industries to seek an environmentally friendly process for the disposal of tobacco waste. The use of TS would not only decrease the soil pollution caused by its improper disposal but also provide additional income for the tobacco producers ([2] #1155). Some progress has been made in the use of TS, such as the extraction of chemicals ([3] #1227), reconstituted tobacco or filler ([4] #1051), and nanofibrillated cellulose ([5] #1210). However, new approaches are necessary for the safe and sustainable use of TS.

Pectinase is widely used in food ([6] #1172), paper, pulp ([7] #1095), and textile industries ([8] #918). This enzyme can be used for the degradation of pectin substances in plant tissues. Several microorganisms, including bacteria and fungi, have been shown to produce pectinase ([9] #1173). To reduce production costs, agro-waste sources, such as fruit waste ([10] #1101; [11] #1092), carrot waste ([12] #1098), and onion wastes ([13] #1211), have been used to produce pectinase using microbial solid-state fermentation. Furthermore, bacterial pectinase is preferred over fungal pectinase because of the ease of fermentation and modern techniques for improving production yield ([11] #1092). In addition, pectinases can be classified into acidic and alkaline enzymes according to the optimal pH for enzymatic activity. Alkaline enzymes, which are almost exclusively produced by bacteria, have numerous applications, such as textile processing; pharmaceutical uses; and leather, detergent and paper production ([6] #1172; [14] #1304).

Based on these criteria, we screened bacteria that could produce pectinase using TS, which is abundant in pectin (approximately 10%) ([15] #1141). Furthermore, the morphological and molecular characteristics of the isolated strain were analyzed. The enzyme properties, production processes with TS, and the purification of the pectinase were studied. This study provides a new strategy for the use of TS and helps lay the foundation for the production of pectinase.

## Results

### Screening of isolates for pectinase activity

**Fig. 1:**
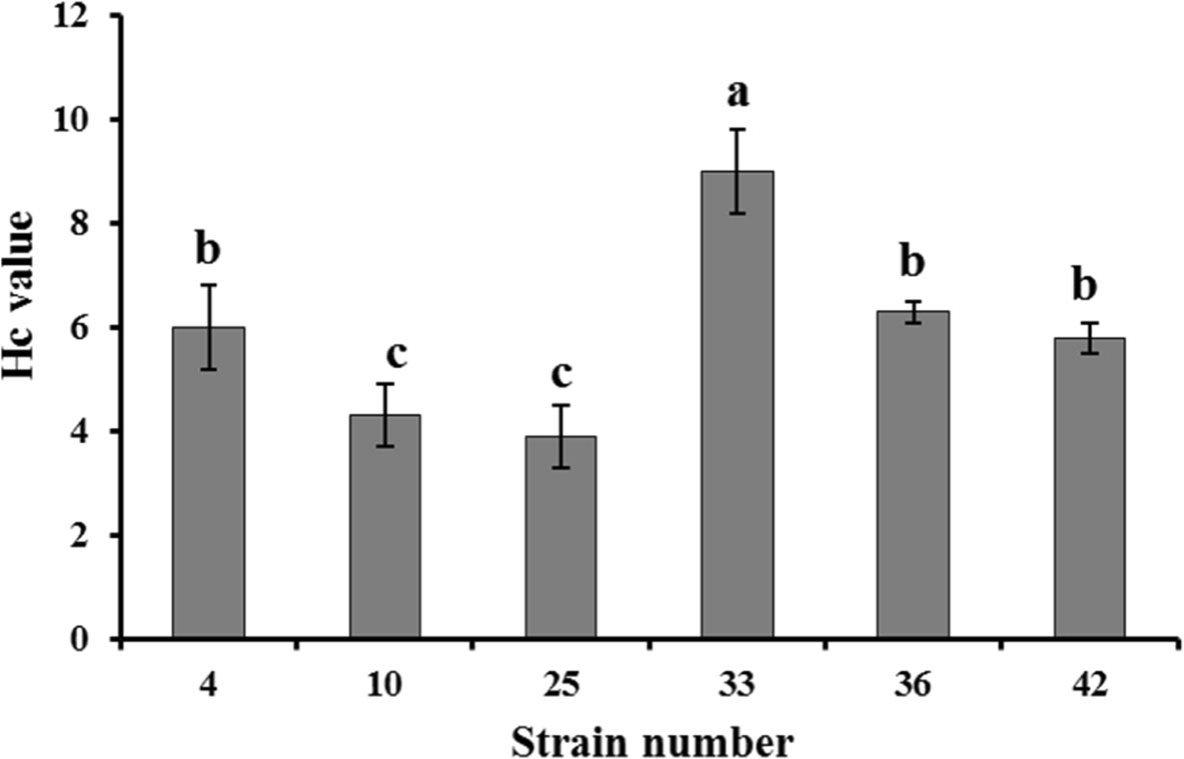
The Hc values of screened strains. Values are given as the means ± standard deviation (*n* = 3). Different letters indicate significant differences at 5%

Pectinase has many applications in various industrial processes ([16] #1170; [17] #1311). To screen excellent strains for pectinase production, some isolates from the primary screening were spot inoculated on pectin agar plates (PAPs) in triplicate. An additional image was shown this in more detail [see Additional file 1: Figure S1]/(see Additional file 1). The Hc value of 33 for this isolate was significantly higher than the values for other isolates (8.87 ± 0.15, Fig. 1). This bacterium was labeled as isolate *B. tequilensis* CAS-MEI-2-33, and further characterization and identification were carried out.

### Morphological characteristics of *B. tequilensis* CAS-MEI-2-33

*B*. *tequilensis* CAS-MEI-2-33 was a gram-positive bacterium that contained spores, and the shape of the bacterial cells was clubbed. When grown at 37 °C for 8–12 h in LB agar, colonies of *B*. *tequilensis* CAS-MEI-2-33 were round, smooth, and cream colored, and the margin was entire.

### Biochemical characterization of *B. tequilensis* CAS-MEI-2-33

The biochemical characterization of *B*. *tequilensis* CAS-MEI-2-33 was performed using a variety of tests, including the Voges–Proskauer, nitrate reduction, glucose utilization, catalase, motility, lysozyme tolerance, phenylalanine, gelatin, starch, lactose, casein, and mannitol tests. The results of the phenylalanine, starch, and mannitol tests were negative, while the results of the remaining tests were positive (Table 1).

**Table 1:** Biochemical characterization of the isolate *B*. *tequilensis* CAS-MEI-2-33. (+, positive; −, negative)

Biochemical test	CAS-MEI-2-33
Voges–Proskauer	+
Nitrate reduction	+
Glucose utilization	+
Catalase	+
Motility	+
Lysozyme tolerance	+
Phenylalanine	–
Gelatin	+
Starch	–
Lactose	+
Casein	+
Mannitol	–

### Identification based on phylogenetic analysis

**Fig. 2:**
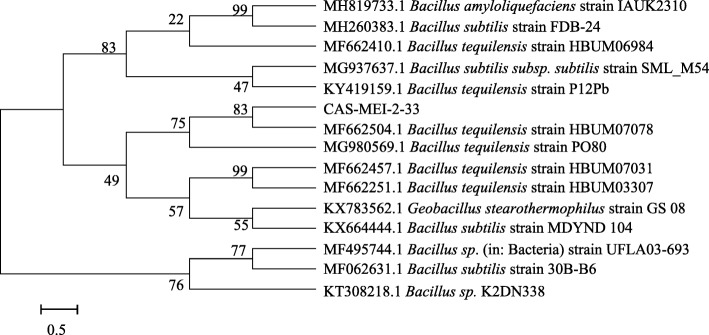
Phylogenetic tree based on 16S ribosomal RNA sequence analysis showing the position of the strain CAS-MEI-2-33 using MEGA 7.0 software. Numbers at branching points refer to bootstrap values (1000 resamplings) with 0.50 as the sequence divergence

The phylogenetic tree generated using the 16S rRNA gene sequence of the bacterial isolate showed the highest homology (100%) with *B*. *tequilensis*. Furthermore, the biochemical characterization of the ability of CAS-MEI-2-33 to metabolize starch and mannitol was different from that of *Bacillus subtilis* but similar to that of *B*. *tequilensis*. According to Bergey’s Manual of Determinative Bacteriology, this strain was named *B*. *tequilensis* CAS-MEI-2-33 ([18] #1309). The constructed phylogenetic tree indicated that this strain had the closest genetic relationship with the *B. tequilensis* strain P12Pb (Fig. 2). The tree was inferred by the neighbor-joining method using MEGA 7.0 software. The numbers at the nodes of the tree are indications of the levels of bootstrap support based on neighbor-joining analysis of 1000 inferred replications.

### Toxicity of nicotine to *B. tequilensis* CAS-MEI-2-33

**Fig. 3:**
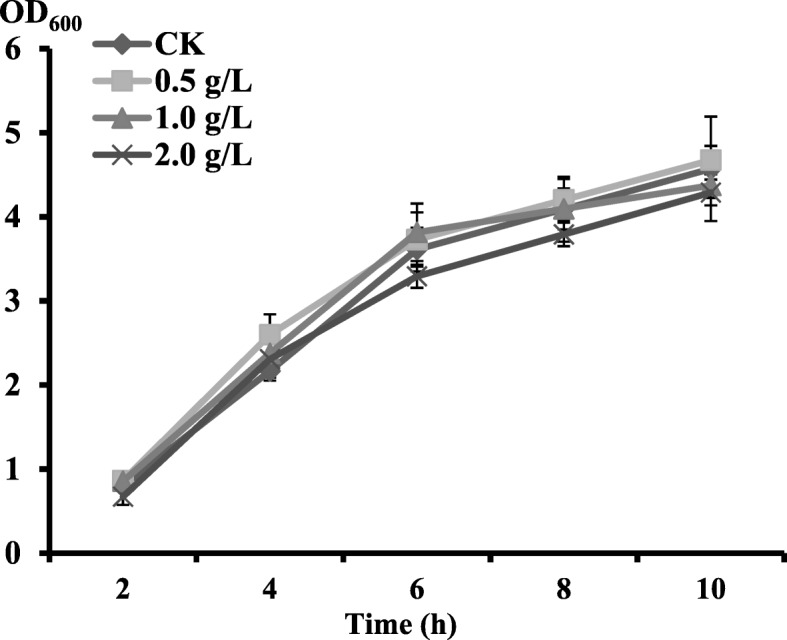
The growth of *B. tequilensis* CAS-MEI-2-33 under different concentrations of exogenous nicotine. Values are given as the means ± standard deviation (n = 3)

TS, contains nicotine, which is harmful to many bacteria, and serves as a distinct source of nutrition. The average nicotine content in TS is as high as 1900–3800 mg/kg ([1] #1136), which is equal to 76–152 mg/L in the fermentation medium. In this study, 500–2000 mg/L nicotine in the medium was tested. The highest concentration of nicotine in the test medium was approximately 13 times that in the TS fermentation medium. However, the growth of *B. tequilensis* CAS-MEI-2-33 was less affected under the experimental conditions (Fig. 3).

### Production of pectinase

**Fig. 4:**
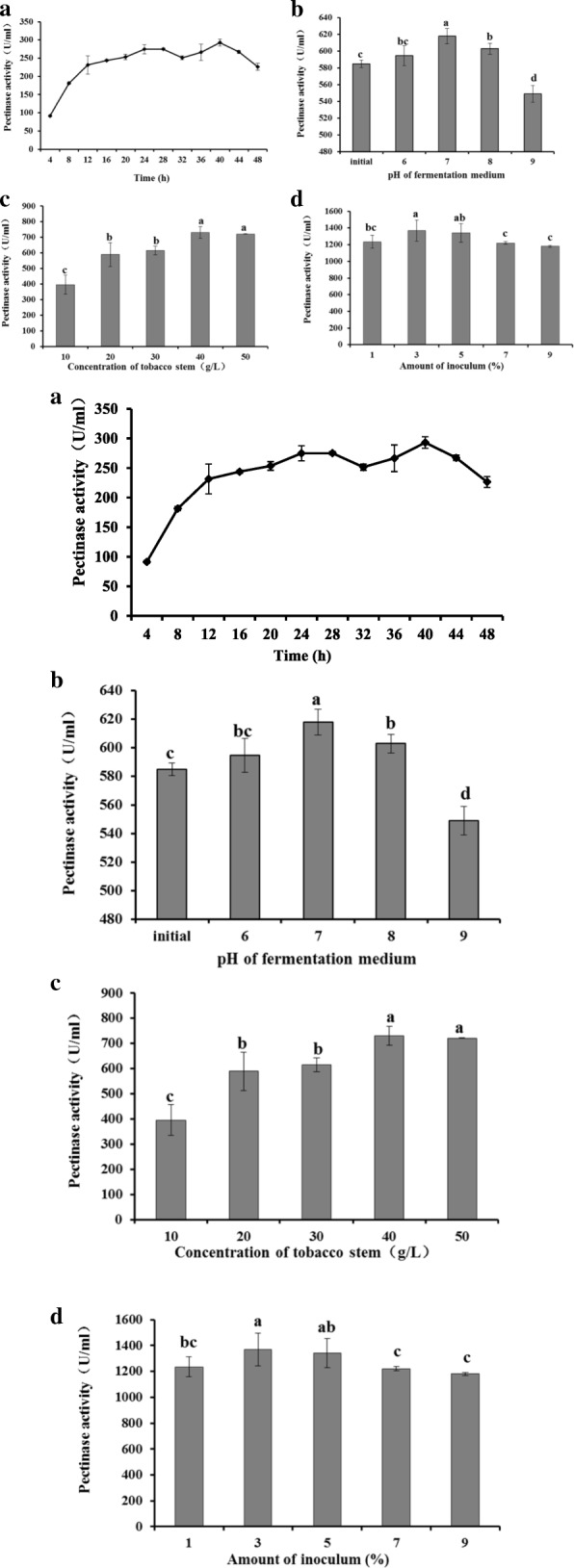
Pectinase activity of *B. tequilensis* CAS-MEI-2-33 during fermentation. **a** Pectinase activity during *B. tequilensis* CAS-MEI-2-33 growth. **b** Effect of the initial pH of fermentation medium on enzyme activity. **c** Effect of tobacco stalk concentration in the fermentation medium on enzyme activity. **d** Effect of the amount of inoculum on enzyme activity. Values are given as the means ± standard deviation (n = 3). Different letters indicate significant differences at 5%

The production of high titers of pectinase by optimizing the growth parameters is of prime importance in industry ([19] #1145). In the present study, the one-factor-at-a-time method was implemented to optimize the components of the medium and conditions. The enzymatic activity was detected using 3,5-dinitrosalicylic acid (DNS) reagent (Beijing Leagene Biotech. Co., Ltd.) based on the production of D-galacturonic acid ([20] #1161). The curve for the production of pectinase during the fermentation of *B*. *tequilensis* CAS-MEI-2-33 was pivotal for indicating the optimal fermentation. The activity of pectinase increased gradually until 40 h, except for 32 h, and then began to decline (Fig. 4a). The highest pectinase activity for the process of fermentation using TS as the feedstock was 293 U/mL at 40 h. Parameters such as the pH of the fermentation medium have a substantial influence on the growth of strains and pectinase production. Pectinase activity (618 ± 9 U/mL, Fig. 4b) was significantly increased at pH 7.0 compared to that under other conditions.

After the determination of pectinase activity with different concentrations of TS in the fermentation medium, it was found that at the concentration of 40 g/L TS and the optimized pH, pectinase activity was the highest (730 ± 38 U/mL, Fig. 4c), as supported by ANOVA (*p* = 0.05).

The inoculum amount played a key role in the initial fermentation. Under the optimal pH and TS concentration, inoculum concentrations of 1, 3, 5, 7, and 9% were tested. The highest pectinase activity (1370 ± 126 U/mL, Fig. 4d) with 3% inoculum was not significantly different from that with 5% inoculum. At higher levels, such as 7 and 9%, enzyme production declined, which could be due to competition for nutrients among the population of bacteria, as has been observed in *Thermomucor indicae-seudaticae* ([21] #1230).

### Pectinase properties

**Fig. 5:**
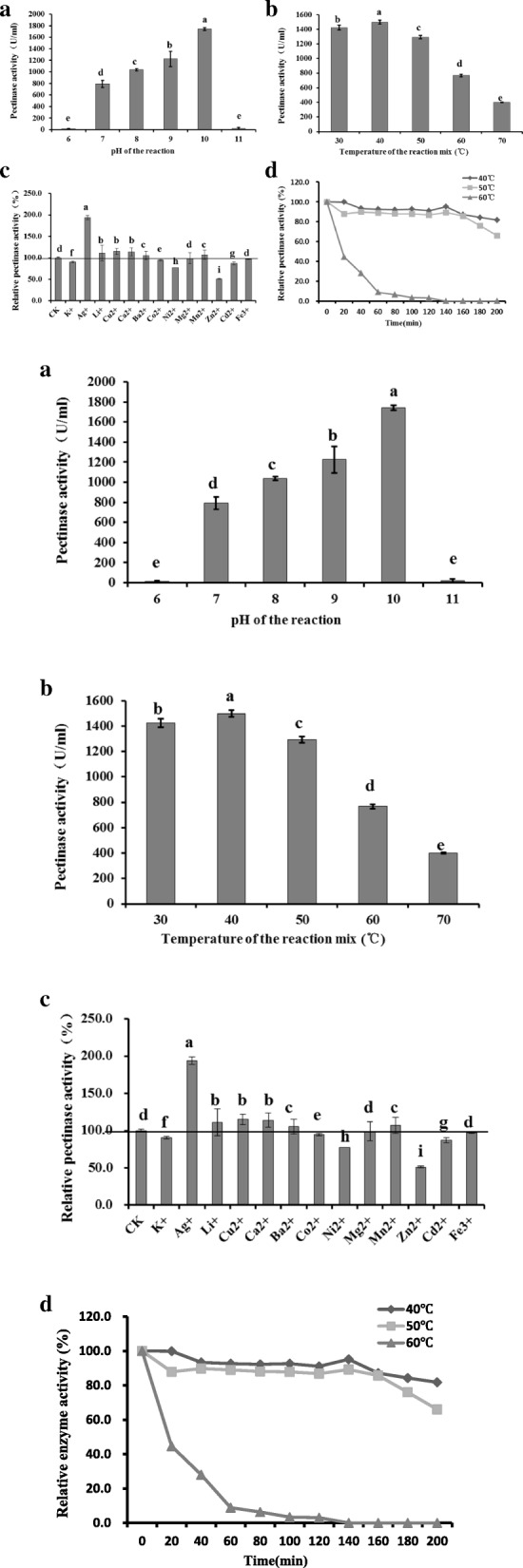
Enzymatic properties of pectinase. **a** Effect of substrate pH on pectinase activity. **b** Effect of reaction temperature on pectinase activity. **c** Effect of metal ions on pectinase activity. **d** Temperature stability of pectinase. Values are given as the means ± standard deviation (n = 3). Different letters indicate significant differences at 5%

The results of the pectinase property analysis are shown in Fig. 5. The pH of the reaction system can affect pectinase activity. Pectinase activity increased when the pH of the reaction systems was increased from 6.0 to 10.0, but this activity was nearly undetectable at pH 11.0. Our results showed that 791 ± 42 U/mL pectinase activity at pH 10.0 was the highest recorded value (Fig. 5a). Subsequently, the influence of temperature on pectinase activity was investigated. Pectinase activity increased when the reaction temperature was increased from 30 °C to 40 °C, reached maximum activity at 40 °C, and then decreased rapidly as the temperature increased beyond 40 °C. The pectinase activity at 40 °C was higher than that at other temperatures (Fig. 5b). Thus, pectinase was more active at an alkaline pH and intermediate temperature. The effects of different metal ions on pectinase activity are shown in Fig. 5c. Ag^+^, Li^+^, Cu^2+^, Ca^2+^, Ba^2+^, and Mn^2+^ ions increased enzyme activity; in particular, Ag^+^ ions increased pectinase activity by 193.95%, which was approximately 1.94 times higher than that of the control. K^+^, Co^2+^, Ni^2+^, Mg^2+^, Zn^2+^, Cd^2+^, and Fe^3+^ ions, especially Zn^2+^, inhibited pectinase activity. Figure 5d shows the thermal stability of pectinase produced by *B*. *tequilensis* CAS-MEI-2-33. However, pectinase activity decreased when the cell-free supernatant was placed at 60 °C. Pectinase activity was stable when the cell-free supernatant was incubated at 40 °C; however, pectinase could not tolerate the high temperature for a long time.

### Partial purification of pectinase from CAS-MEI-2-33

Under the optimal fermentation conditions, 2.30 L supernatant was obtained by centrifuging the bacteria in a 3.0 L fermentation flask. The pectinase activity reached 1771 U/mL, and the total pectinase activity was 4,074,513 U (Table 2).

**Table 2:** The partial purification results of alkaline pectinase

	Total activity (U)	Total protein (ug)	Specific activity	Purification fold
Fermentation liquid	4,074,513	7,859,964	0.5	1
Ammonium sulfate	284,144	166,842	1.7	3.3
High-Q-8.0	77,611	15,066	5.2	10.2
Sephacryl S-100	41,357	1580	26.2	51.4

**Fig. 6:**
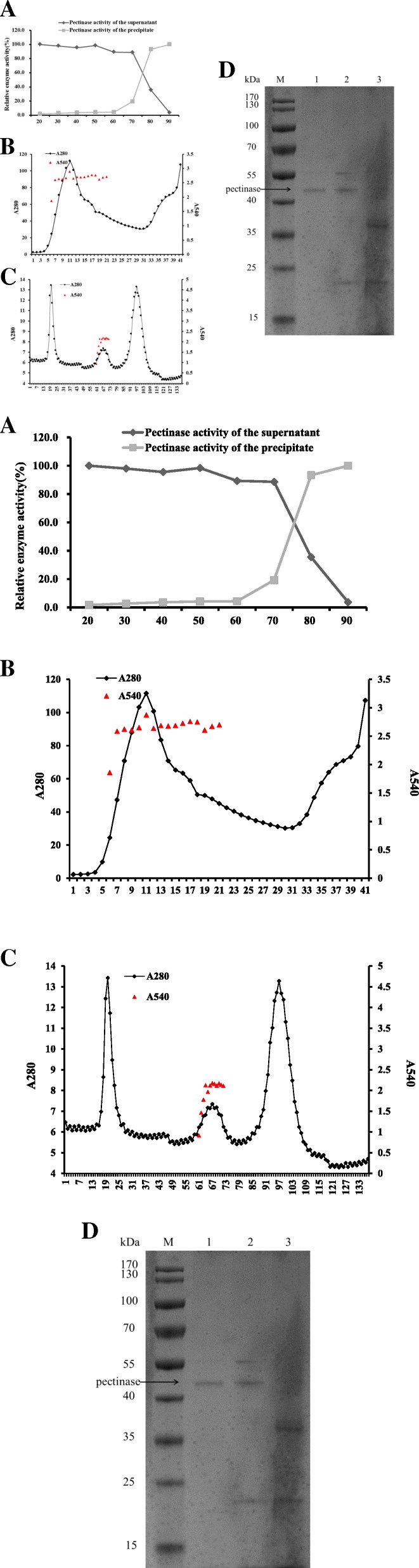
The purification of alkaline pectinase from *B. tequilensis* CAS-MEI-2-33. **a** Ammonium sulfate fractionation curve; **b** Elution curve of Mini Macro-Prep High-Q ion exchange chromatography; **c**. Elution curve of Sephacryl S-100 column chromatography; **d**. The partial purification of alkaline pectinase from *B*. *tequilensis* CAS-MEI-2-33 using TS. M: molecular weight makers; 1. Sephacryl S-100 column chromatography with pH 7.2; 2: Mini Macro-Prep High-Q ion exchange chromatography with pH 8.0; 3. Concentrated ammonium sulfate salting out solution

According to the ammonium sulfate fractionation curve, suitable saturation was selected for salting out. The fractionation curve is shown in Fig. 6a. When ammonium sulfate was saturated to 70–80%, the pectinase activity of the precipitate increased, while that of the supernatant decreased significantly. When the saturation rate reached 80–90%, the pectinase activities (pH 10.0, Gly-NaOH) of the precipitate and supernatant did not change significantly. The target protein was dissolved with 30 mL buffer (pH 8.0, Tris-HCl) after salting out. Then, the target protein was further renatured with 55.0 mL buffer (pH 8.0, Tris-HCl) through a dialysis bag. The pectinase activity was 5166 U/mL, and the total activity was 284,144 U.

After dialysis, the supernatant was purified with a Mini Macro-Prep High-Q ion exchange column. The results are shown in Fig. 6b. The pectinase was eluted with pH 8.0 Tris-HCl buffer, and when the column was gradiently eluted with pH 8.0 Tris-HCl buffer containing 0–1 mol/L NaCl, peaks eluted. By detecting pectinase activity, it was found that the first elution peak was active. The first elution peak was collected with pectinase activity of 862,352 U/mL. The total activity was 77,611 U. Sephacryl S-100 was equilibrated with ultrapure water, sampled, and then eluted with pH 7.2 PBS buffer, with a flow rate of 0.8 mL/min, and the effluent was detected online by a UV detector at a wavelength of 280 nm to record the ultraviolet absorption peak curve (Fig. 6c). The components were collected and used for the determination of enzyme activity and protein content. The pectinase activity was 13,786 U/mL, and the total activity was 41,357 U.

**Fig. 7:**
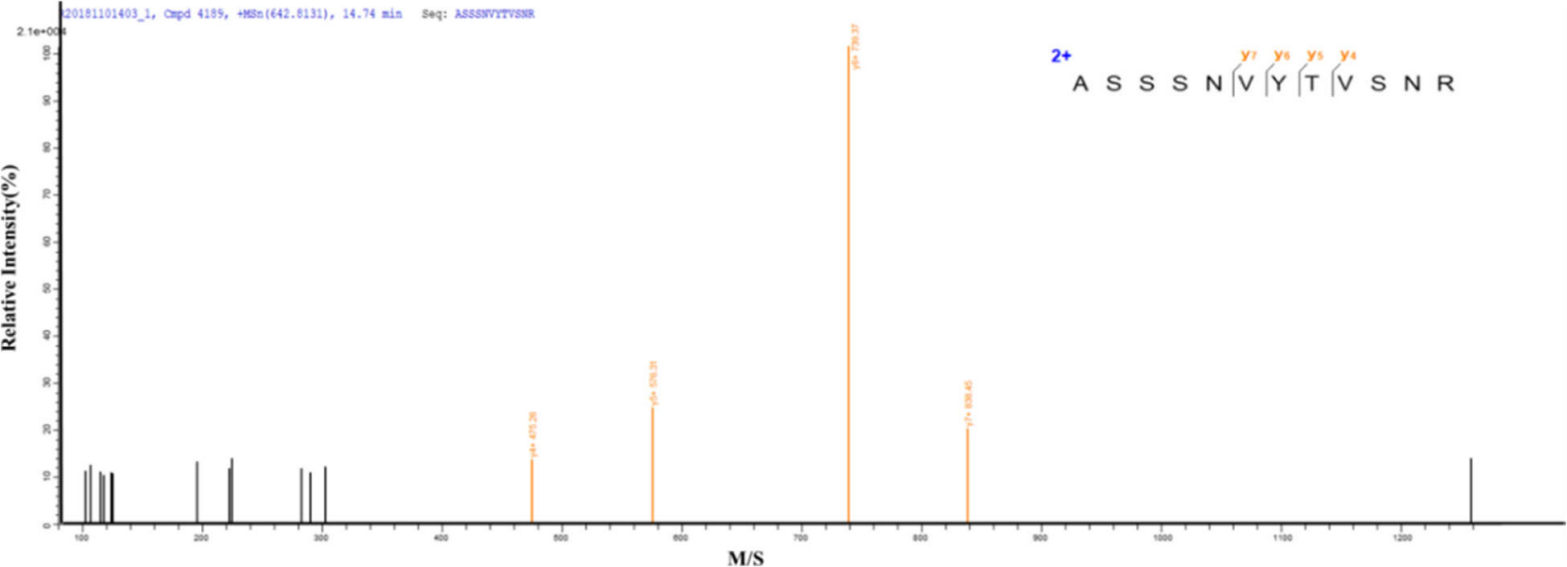
LC-MS/MS analysis of protein bands by SDS-PAGE

The molecular weight of the purified alkaline pectinase was detected with SDS-polyacrylamide gel electrophoresis (PAGE) as described by Mehrnoush et al. ([22] #1306). The molecular weight of the pectinase, which was approximately 45.4 kDa, is shown in Fig. 6d. Then, the protein band was cut from the SDS-PAGE gel, subjected to LC-MS/MS analysis by Shanghai Applied Protein Technology ([23] #1320; [24] #1321), and identified by searching the UniProt database. The additional figure about protein base peak was shown this in more detail [see Additional file 2: Figure S2]/(see Additional file 2 for protein peak). Finally, the protein was identified as pectate lyase, the sequence was K.ASSSNVYTVSNR.N (Fig. 7). The molecular weight was 45.4 kDa, which was consistent with the SDS-PAGE results. The results indicated that the enzyme was a good candidate for pectate lyase. Furthermore, this study was a new attempt to recycle and reuse TS in agricultural production.

## Discussion

This study made a successful primary attempt to produce pectinase using TS with a new strain. In previous studies, a variety of microorganisms were identified as pectinase producing, including *B. subtilis* ([8] #918; [25] #921), *B. megaterium* ([26] #1152), *Rhizopus oryzae* ([27] #1147), *Aspergillus oryzae* ([10] #1101), and *B. tequilensis* ([28] #1307).

In addition, it was critical to determine the fermentation period of *B*. *tequilensis* CAS-MEI-2-33 during optimization experiments. The one-factor-at-a-time method was implemented to optimize the components of the medium and conditions, especial the pH of medium. The optimum pH for the growth of and pectinase production by other bacterial strains, such as *B. subtilis* ([8] #918), has been recorded to vary from 7.0 to 10.0. Meneghel et al. ([29] #1193) and Fawole and Odunfa ([30] #1231) have reported that pectinase production by *Aspergillus* fungi occurs at an acidic pH. Different strains have species-specific acid-base conditions for their growth and fermentation processes. And then, as the primary carbon source in the medium, the concentration of TS exerted a profound effect on pectinase production by *B*. *tequilensis* CAS-MEI-2-33. However, pectinase activity with 50 g/L TS in the fermentation medium was not significantly different from that with 40 g/L TS, and this similarity might be caused by the other components, such as tobacco-specific nitrosamines (TSNAs) ([31] #1310).In the optimized fermentation (40 h, pH 7.0, 40 g/L TS, 3% inoculum), the highest pectinase activity was 1370 U/ml. By optimizing the inoculum amount, pectinase activity was improved 1.88 times. The results indicated that the *B. tequilensis* CAS-MEI-2-33 strain was a good candidate for biorefining TS. In subsequent experiments, optimization to improve the efficiency of pectinase production using TS and exploration of applications in industry, as conducted by Swarupa Rani Chiliveri ([28] #1307), would attract more interest.

The properties of pectinase are important in the application of this enzyme ([16] #1170); in particular, enzyme deactivation and stability are considered major constraints. The pectinase enzymes from different strains exhibited different properties ([16] #1170). In this study, the enzyme was an alkaline pectinase, which has various environmental and economic applications, such as textile processing; pharmaceutical uses; and leather, detergent and paper industry ([6] #1172). Pectinase is produced by both prokaryotic microorganisms, which almost exclusively synthesize alkaline pectinases, and eukaryotic microorganisms, which synthesize acid pectinases ([6] #1172). Ping & Chaochao reported that the optimal pH for pectinase production by *Bacillus* sp. was 5.0 ([32] #1221). The difference in optimal pH might be caused by the strain or fermentation medium. This is the first report of the ability of *B*. *tequilensis* strain to produce pectinase using TS and provides a direction for further investigation of the possible applications of TS.

## Conclusions

This study successfully determined that the strain isolated from the cigar wrapper was a potential candidate organism that was useful in the production of pectinase using TS. The *B*. *tequilensis* CAS-MEI-2-33 strain will not only aid in addressing TS waste but also increase the possible sources of alkaline pectinase. The medium and culture conditions for this strain were optimized in this study. Based on the optimized conditions (pH 7.0, 40 g/L TS, 3% inoculation, and 40 h cultivation), the highest enzymatic activity of the strain was 1370 U/mL. The enzymatic properties were studied, including the pH of the reaction buffer, reaction temperature, effects of metal ions, and thermal stability. The alkaline pectinase was relatively stable at 40 °C, and the molecular weight was approximately 45.4 kDa. This is the first report of the ability of the *B*. *tequilensis* strain to produce pectinase using TS and provides a direction for further investigation of the possible applications of TS.

## Methods

### Primary screening

Samples collected from the cigar wrapper leaf from the Haikou Cigar Research Institute, Hainan Provincial Branch of the China National Tobacco Corporation, were diluted with 0.9% sterile NaCl solution and then plated onto pectin agar plates (PAPs) containing 0.5% pectin (galacturonic acid≥ ≥ 74.0%, Shanghai Macklin Biochemical Co., Ltd.), 0.1% K_2_HPO_4_, 0.3% (NH_4_)_2_SO_4_, 0.001% Fe_2_SO_4_, 0.1% MgSO_4_, and 1.8% agar powder. Inoculated plates were incubated for 48 h at 37 °C to obtain bacterial colonies. Colonies were picked, inoculated on fresh PAPs, and incubated at 37 °C for 48 h to obtain pure cultures. The pure cultures were maintained on Luria–Bertani (LB) agar plates at 4 °C and stored at − 80 °C with 15% glycerol for further use.

### Secondary screening

The bacterial isolates from the primary screening were tested for their ability to produce pectinases. The isolates were spot inoculated on PAPs in triplicate and incubated at 37 °C for 36–48 h. Depending on the zone of clearance surrounding the colonies (Hc value, the ratio of the diameter between clearance and the strain) using Congo red (Shanghai Aladdin Bio-Chem Technology Co., Ltd.) ([10] #1101), the strain used for further experiments was selected.

### Identification of the isolated bacterial strain

The isolated bacterial strain was identified according to morphological, physiological, and biochemical characteristics, as well as phylogenetic analysis of the 16S ribosomal RNA (rRNA) gene. Morphological characteristics of the isolated bacterial strain, e.g., colony morphology (color, shape, margin, and surface) and cell morphology (shape and Gram staining), were studied ([25] #921). The biochemical characteristics of the strain were studied with microbial biochemical identification tubes (Guangdong Huankai Microbial Sci. & Tech. Co., Ltd.). These biochemical and characterization tests include the Voges–Proskauer, nitrate reduction, glucose utilization, catalase, motility, lysozyme tolerance, phenylalanine, gelatin, starch, lactose, casein, and mannitol tests. The 16S rRNA gene fragment of the strain was obtained using polymerase chain reaction (PCR) amplification with universal primers (BGI Life Tech Co., Ltd., Qingdao, China): 27F (5′-3′): AGAGTTTGATCCTGGCTCAG and 1492R (5′-3′): ACGGCTACCTTGTTACGACTT. The reaction mixture was prepared in a final reaction volume of 50 μl consisting of 25.0 μl Premix Taq™ (LA Taq™ Version 2.0, Takara Bio, Japan), 1.0 μl 10 μM primers, 1.0 μl genomic DNA template (57 ng/μl), and 22.0 μl ddH_2_O. The genomic DNA of *B*. *tequilensis* CAS-MEI-2-33 was extracted with a TIANamp Bacteria DNA Kit (TIANGEN Biotech, Beijing) according to the manufacturer’s instructions. The PCR products were detected using 1.0% agarose gel electrophoresis. The product of the appropriate size (1.5 kbp) was sequenced (BGI Life Tech, China), and the sequence was compared with the NCBI nucleotide sequence database (https://blast.ncbi.nlm.nih.gov/Blast.cgi?PROGRAM=blastn&PAGE_TYPE=BlastSearch&LINK_LOC=blasthome) and deposited under accession number MH806356.1. The phylogenetic tree was constructed using MEGA (version 7) ([33] #1324). The phylogenetic tree was inferred using the neighbor-joining method. Bootstrap analysis was based on 1000 resamplings.

### Pectinase activity assay

Pectinase activity production was assessed using the 3,5-dinitrosalicylic acid (DNS) method ([29] #1193; [9] #1173), and the absorbance was measured at 540 nm with a UV-VIS spectrophotometer (Cary 50 UV-Vis, Varian, Inc., North America). Cell-free supernatant (20 μl) was mixed with 1 mL pectin (0.2%, galacturonic acid≥74.0%, Shanghai Macklin Biochemical Co., Ltd.) as the substrate using NaOH-glycine buffer (50 mmol/L, pH 9.0) and incubated at 40 °C for 30 min. DNS solution (1 mL; Beijing Leagene Biotechnology Co., Ltd., China) was added, and the reaction mixture was boiled for 5 min. The absorbance of the cooled reaction mixture was read at 540 nm, while the supernatant was boiled for 5 min to inactivate the pectinase.

A standard curve was established using D-galacturonic acid as the reducing sugar. The final concentrations of the solutions in each tube with 5 mL DNS solution were 0.008, 0.016, 0.024, 0.032, 0.040, and 0.048 mg/mL D-galacturonic acid (Beijing Leagene Biotechnology Co., Ltd.). The tubes were boiled for 5 min and cooled. A blank sample was prepared with distilled water instead of D-galacturonic acid. One unit (U) of polygalacturonase activity was defined as the amount of enzyme that generated 1 μmol galacturonic acid per min under the assay conditions. Pectinase activity was calculated using the following formula:

$$ \mathrm{Enzyme}\kern0.17em \mathrm{A}\mathrm{ctivity}\;\left(\frac{\mathrm{U}}{\mathrm{ml}}\right)=\frac{1000\times \Delta \mathrm{A}\times \mathrm{N}}{\mathrm{K}\times \mathrm{M}\times \mathrm{T}} $$

where ΔA is the absorbance of samples subtracted from control absorbance, N is the dilution factor, K is the slope of the standard curve, T is 30 min, and M is the molecular weight of D-galacturonic acid (M = 194.14 Da).

### Optimization of the fermentation conditions

It is critical to determine the fermentation period of *B*. *tequilensis* CAS-MEI-2-33 during optimization experiments. We took samples and detected the pectinase activity of the fermentation medium every 4 h to confirm the maximum activity of pectinase during fermentation. The initial fermentation medium contained 20 g/L TS, 0.5 g/L MgSO_4_, 3 g/L (NH_4_)_2_SO_4_, and 0.01 g/L Fe_2_SO_4_ at pH 7.0. Fermentation was performed in shake-flask experiments using 500 mL shake flasks containing 100 mL fermentation medium incubated with the strain *B*. *tequilensis* CAS-MEI-2-33. The culture was grown at 37 °C and 180 rpm. All experiments were performed in triplicate. The reagents were purchased from Sinopharm Chemical Reagent Co., Ltd.

Inocula were prepared using the culture of *B*. *tequilensis* CAS-MEI-2-33 incubated for 8 h at 37 °C in LB. The OD_600_ of *B*. *tequilensis* CAS-MEI-2-33 was approximately 4.52, and the inoculum volume was 1% of the fermentation volume. All experiments were performed in triplicate. One-way ANOVA with Tukey’s test was used to determine any significant differences (*p* < 0.05) between treatments using SAS (Statistical Analysis System, SAS Institute Inc., USA).

To test the toxicity of nicotine to *B. tequilensis* CAS-MEI-2-33, growth of this strain in LB containing nicotine was evaluated in shake-flask experiments using 150 mL shake flasks containing 50 mL medium that was incubated with 1% (v/v) inocula. Nicotine was added to the LB medium at final concentrations of 0, 0.5, 1.0, and 2.0 g/L. The optical density of the incubation was monitored at 600 nm every two hours.

Optimization of the pH was performed in a similar manner for the selected isolate. The initial fermentation medium was prepared with pH ranging from 6.0–8.0 using NaOH and the initial medium prior to sterilization at 121 °C for 20 min. The medium was incubated at 37 °C and 180 rpm for 40 h.

TS provided the carbon and nitrogen sources in the medium and was important during fermentation. The fermentation medium was prepared with TS concentrations ranging from 10 to 50 g/L at the optimal pH. The medium was incubated at 37 °C and 180 rpm for 40 h.

Effect of the inoculum size on enzyme production was studied with different concentrations of inocula. Different amounts, i.e., 1, 3, 5, 7, and 9% (v/v), were inoculated into the fermentation medium with 40 g/L TS at pH 7.0. The medium was incubated at 37 °C and 180 rpm for 40 h.

### Enzymatic properties

The enzyme activity was highly influenced by the pH, temperature, and metal ions. The pH and temperature are important characteristics of a biocatalyst for use in industrial applications. The optimal pH of pectinase in the cell-free supernatant was assayed using citrate–phosphate (pH 6.0), phosphate (pH 7.0–8.0), and glycine–NaOH (pH 9.0–11.0) as buffers (50 mmol/L) and 0.2% pectin (Shanghai Macklin Biochemical Co., Ltd., China) as a substrate at 40 °C. The optimal temperature for pectinase activity was determined by incubating the reaction mixture from 30 °C to 70 °C at the optimal pH. The effects of metal ions (Ag^+^, K^+^, Li^+^, Cu^2+^, Ca^2+^, Ba^2+^, Co^2+^, Ni^2+^, Mg^2+^, Mn^2+^, Zn^2+^, Cd^2+^, and Fe^3+^) on pectinase activity were assayed at a concentration of 1.0 mM in the reaction mixture. Thermal stability was investigated by measuring pectinase activity every 20 min following incubation of the enzyme solution at 40 °C, 50 °C, and 60 °C.

### Purification of pectinase

According to the optimal fermentation parameter, we obtained approximately 2.30 L fermentation liquid. After fermentation, the fermentation liquid was centrifuged at 4 °C and 5000×g for 30 min to collect the supernatant, which contained the alkaline pectinase. Crude protein was obtained after separation with ammonium sulfate fractionation, ion exchange chromatography and column chromatography.

Fifty milliliters of supernatant was placed in an ice bath, and 20, 30, 40, 50, 60, 70, 80, and 90% saturation with ammonium sulfate was conducted overnight for protein precipitation. Then, the solution was centrifuged in a high-speed refrigerated centrifuge at 5000×g for 15 min to obtain the precipitate and supernatant. By detecting the pectinase activity of the precipitate and supernatant with different levels of saturation with ammonium sulfate, the ammonium sulfate fractionation curve and suitable saturation were determined. Based on the results from the ammonium sulfate fractionation curve, 70% saturation with ammonium sulfate was conducted, and the precipitate was removed by centrifugation. Then, the supernatant was brought to 80% saturation with ammonium sulfate to precipitate the target enzyme. The precipitate was crude pectinase. By dialyzing the precipitate a few times in equilibrated buffer, the crude enzyme was obtained.

The crude enzyme was subjected to High-Q ion exchange chromatography (Bio-Rad Laboratories (Shanghai Co., Ltd.)). Then, the column was equilibrated with Tris-HCl buffer (pH 8.0) and eluted with a linear salt gradient (0–1 M NaCl in 200 mL Tris-HCl buffer, 0.4 mM, pH 8.0). The eluent was collected automatically at 1 min/tube.

Sephacryl S-100 was equilibrated with ultrapure water, sampled, and eluted with pH 7.2 PBS buffer, with a flow rate of 0.8 mL/min, and the effluent was detected by an online UV detector at a wavelength of 280 nm to record the ultraviolet absorption peak curve. The components were collected, and the enzyme activity and protein content were tested.

### Determination of protein content and SDS-polyacrylamide gel electrophoresis (SDS-PAGE)

The protein content was determined using a BCA (bovine serum albumin) standard curve, and SDS-PAGE ([34] #1297) was conducted to identify the enzyme purity.

### LC-MS/MS identification of protein

Protein bands were cut from the SDS-PAGE gel and subjected to LC-MS/MS (liquid chromatographic-tandem mass spectrometric) analysis by Shanghai Applied Protein Technology ([23] #1320; [24] #1321). Protein samples were digested using trypsin for 20 h at 37 °C. Then, peptides were trapped and desalted on Zorbax 300SB-C18 peptide traps (Agilent Technologies, Wilmington, DE, USA) followed by separation on a C18-reversed-phase column (0.15 × 150 mm, Column Technology Inc., Fremont, CA, USA). An Easy nLC system (Thermo Fisher Scientific) was used to deliver mobile phases A (0.1% formic acid) and B (0.1% formic acid in 84% acetonitrile) ([35] #1322) according to routine methods. The mass spectrometer was operated in MS/MS mode scanning from 380 to 1800 amu. The top 20 multiply charged ions were selected from each scan for MS/MS analysis ([36] #1323). Then, the MS/MS spectra were searched using MASCOT 2.2 (Matrix Science, London, UK), and the protein was identified by searching the *B*. *tequilensis* database in UniProt (https://www.uniprot.org/).

## Additional files

Additional file 1: **Figure S1.** The transparent zone around the strain of *B. tequilensis* CAS-MEI-2-33 on PAPs. (DOCX 65 kb)

Additional file 2: **Figure S2.** Protein peak detected by IC-MS/MS. The protein was isolated from a gel. (DOCX 78 kb)

## Abbreviations

16S rRNA: 16S ribosomal RNA
ANOVA: Analysis of variance
*B*. *tequilensis*: *Bacillus tequilensis*
DNS method: 3,5-dinitrosalicylic acid method
LB: Luria–Bertani
LC-MS/MS: Liquid chromatographic-tandem mass spectrometric
PAPs: Pectin agar plates
PCR: Polymerase chain reaction
TS: Tobacco stalk
