# Supplementary material for: Production of alkaline pectinase: a case study investigating the use of tobacco stalk with the newly isolated strain Bacillus tequilensis CAS-MEI-2-33
Source: BMC Biotechnol. 2019 Jul 12;19:45. doi: 10.1186/s12896-019-0526-6 (PMC6624900; doi:10.1186/s12896-019-0526-6)
Supplement: Supplementary file 1 — Figure S1. The transparent zone around the strain of B. tequilensis CAS-MEI-2-33 on PAPs. (DOCX 65 kb) [file 12896_2019_526_MOESM1_ESM.docx]

**Additional file 1**


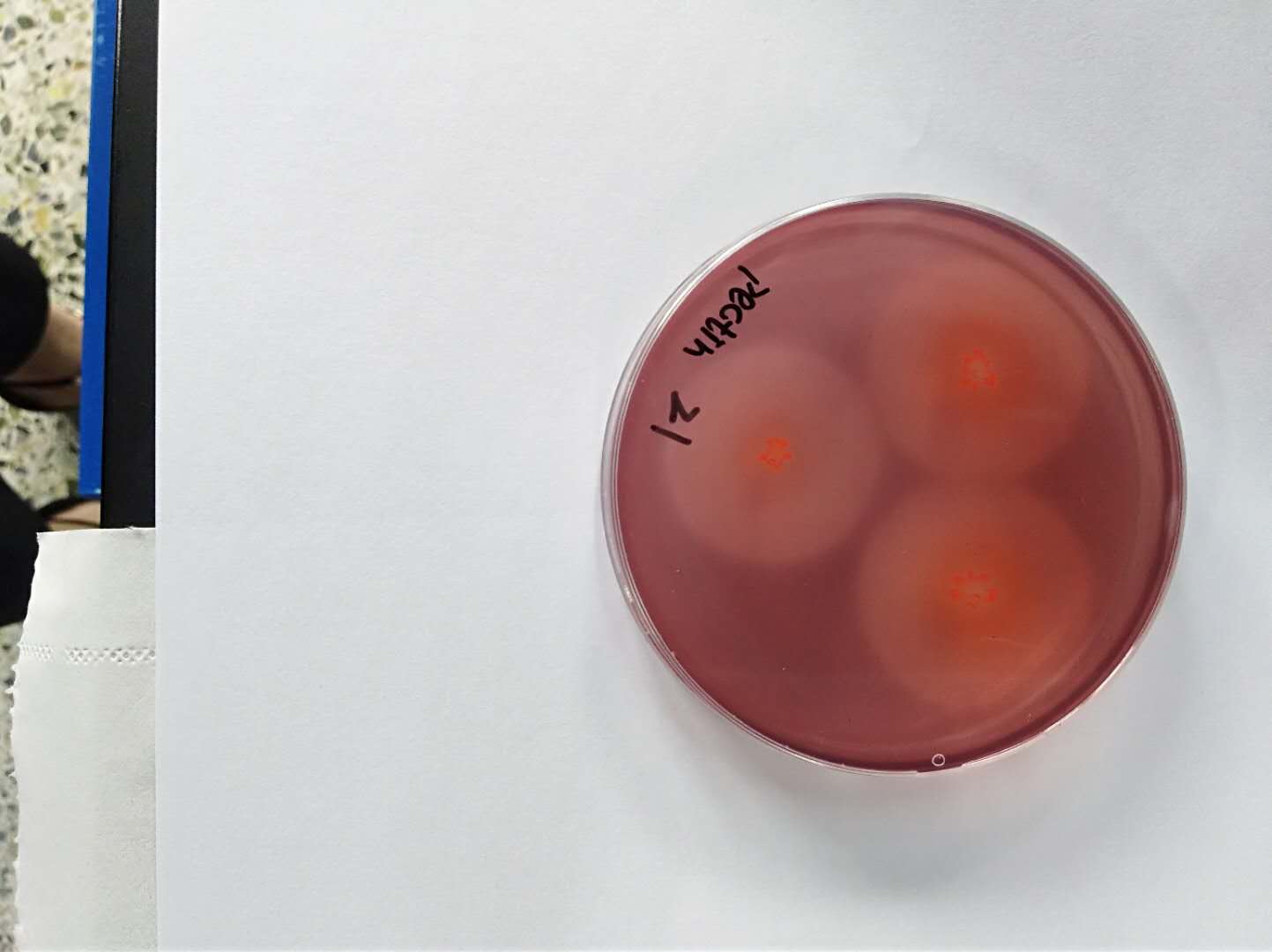


Figure S1. The transparent zone around the strain of *B. tequilensis* CAS-MEI-2-33 on PAPs.
