# Supplementary figures and images for: Production of alkaline pectinase: a case study investigating the use of tobacco stalk with the newly isolated strain Bacillus tequilensis CAS-MEI-2-33
Source: BMC Biotechnol. 2019 Jul 12;19:45. doi: 10.1186/s12896-019-0526-6 (PMC6624900; doi:10.1186/s12896-019-0526-6)

**Additional file 2**


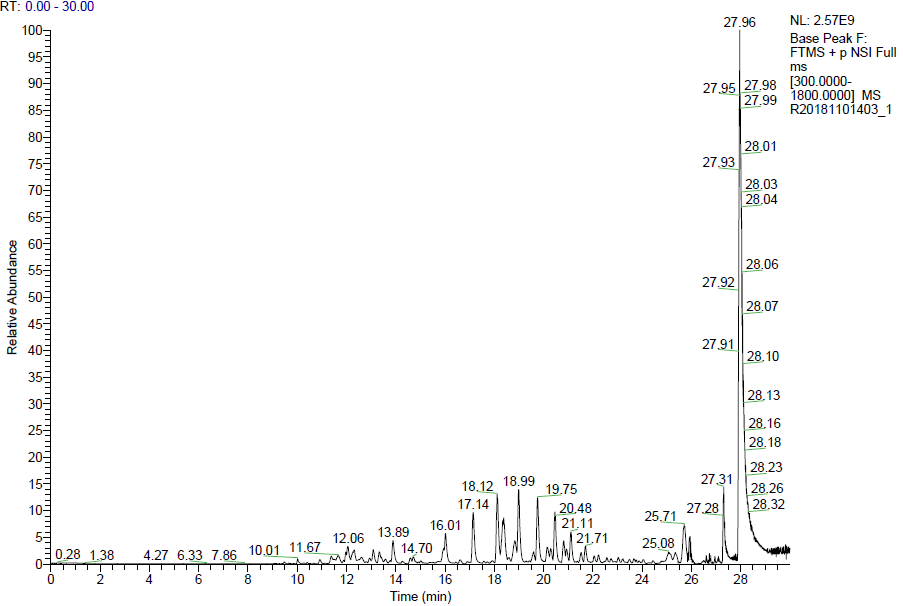


Figure S2. Protein peak detected by IC-MS/MS. The protein was isolated from a gel.

Supplement: Supplementary file 2 — Figure S2. Protein peak detected by IC-MS/MS. The protein was isolated from a gel. (DOCX 78 kb) [file 12896_2019_526_MOESM2_ESM.docx]
